# Novel Classes and Evolutionary Turnover of Histone H2B Variants in the Mammalian Germline

**DOI:** 10.1093/molbev/msac019

**Published:** 2022-01-31

**Authors:** Pravrutha Raman, Mary C Rominger, Janet M Young, Antoine Molaro, Toshio Tsukiyama, Harmit S Malik

**Affiliations:** 1 Division of Basic Sciences, Fred Hutchinson Cancer Research Center, Seattle, WA, USA; 2 Whitman College, Walla Walla, WA, USA; 3 Genetics, Reproduction and Development (GReD) Institute, CNRS UMR 6293, INSERM U1103, Université Clermont Auvergne, Clermont-Ferrand, France; 4 Howard Hughes Medical Institute, Fred Hutchinson Cancer Research Center, Seattle, WA, USA

**Keywords:** histone variants, gene duplication, pseudogenes, positive selection, oogenesis, spermatogenesis

## Abstract

Histones and their posttranslational modifications facilitate diverse chromatin functions in eukaryotes. Core histones (H2A, H2B, H3, and H4) package genomes after DNA replication. In contrast, variant histones promote specialized chromatin functions, including DNA repair, genome stability, and epigenetic inheritance. Previous studies have identified only a few H2B variants in animals; their roles and evolutionary origins remain largely unknown. Here, using phylogenomic analyses, we reveal the presence of five H2B variants broadly present in mammalian genomes. Three of these variants have been previously described: H2B.1, H2B.L (also called subH2B), and H2B.W. In addition, we identify and describe two new variants: H2B.K and H2B.N. Four of these variants originated in mammals, whereas H2B.K arose prior to the last common ancestor of bony vertebrates. We find that though H2B variants are subject to high gene turnover, most are broadly retained in mammals, including humans. Despite an overall signature of purifying selection, H2B variants evolve more rapidly than core H2B with considerable divergence in sequence and length. All five H2B variants are expressed in the germline. H2B.K and H2B.N are predominantly expressed in oocytes, an atypical expression site for mammalian histone variants. Our findings suggest that H2B variants likely encode potentially redundant but vital functions via unusual chromatin packaging or nonchromatin functions in mammalian germline cells. Our discovery of novel histone variants highlights the advantages of comprehensive phylogenomic analyses and provides unique opportunities to study how innovations in chromatin function evolve.

## Introduction

The genome and epigenome together determine form and function in all organisms. A significant component of the epigenome in eukaryotes comprises DNA-packaging units called nucleosomes. Eukaryotic nucleosomes typically contain ∼147 bp of DNA spooled around an octamer of four core histones—H2A, H2B, H3, and H4 (Kornberg 1974; Kornberg and Thomas 1974; Thomas and Kornberg 1975; Luger et al. 1997). These histone proteins share ancestry with histones from Archaea (Pereira et al. 1997; Reeve et al. 1997; Sandman and Reeve 2000; Ammar et al. 2012; Mattiroli et al. 2017; Talbert et al. 2019) and some giant viruses (Erives 2017; Yoshikawa et al. 2019; Liu et al. 2021; Valencia-Sánchez et al. 2021). All histone proteins possess a conserved histone fold domain (HFD) and more divergent N- and C-terminal tails. In eukaryotes, the core histones are typically expressed during genome replication with peak expression in S phase, to repackage newly replicated genomes (Talbert and Henikoff 2021). Hence, they are also called replication-coupled (or RC) histones.

In addition to RC histones, eukaryotes encode histone variants to promote functional diversity and specificity in cellular processes. Histone variants are commonly expressed throughout the cell cycle. As a result, they are also referred to as replication-independent (or RI) histones. RI or variant histones replace RC histones in nucleosomes to promote specialized functions like DNA repair, chromosome segregation, and gene regulation (Talbert and Henikoff 2010; Martire and Banaszynski 2020). Typically, RC histones are present in eukaryotic genomes in large multicopy arrays whereas histone variants are found in one or a few copies. Crucial differences in their HFDs distinguish the sequence and function of histone variants from their RC histone counterparts. In addition, histone variants often significantly differ from RC histones in their N- and C-terminal tails. These differences lead to their deposition by different chaperones and distinct posttranslational modifications, thus resulting in specialized functions by altering chromatin properties (Bönisch and Hake 2012; Henikoff and Smith 2015; Talbert and Henikoff 2017; Molaro et al. 2018).

Some histone variants arose in early eukaryotic evolution, whereas others have evolved more recently in specific lineages (Malik and Henikoff 2003; Talbert and Henikoff 2010). Examples of ancient, well-conserved histone variants include H2A.Z, often found at transcription start sites, and CenH3, which localizes to centromeric DNA across most eukaryotes. However, other histone variants have evolved more recently in specific lineages (Eirín-López et al. 2004; Yelagandula et al. 2014; Rivera-Casas et al. 2016; Molaro et al. 2018). Many H2A variants, including macroH2A, H2A.W, and “short” H2A variants, are found exclusively in filozoans (choanoflagellates and animals), plants, and placental mammals, respectively (Yelagandula et al. 2014; Kawashima et al. 2015; Rivera-Casas et al. 2016; Molaro et al. 2018). Although most eukaryotic histone variants appear to evolve under strong purifying selection, CenH3 also evolves adaptively in plant and animal lineages. CenH3’s rapid evolution has been proposed to be due to centromere drive or competition during female meiosis (Henikoff et al. 2001; Malik and Henikoff 2009). Like ancient histone variants, most lineage-specific histone variants also evolve under strong purifying selection. However, some, like H2A.W in plants (Kawashima et al. 2015) and short H2A variants in mammals (Molaro et al. 2018), show signatures of adaptive evolution. Furthermore, whereas most histones are ubiquitously expressed, many lineage-specific histones, including some plant H2A.W variants and short H2A variants in mammals, are predominantly expressed in germ cells (Govin et al. 2007; Boussouar et al. 2008; Ferguson et al. 2009; Molaro et al. 2018; Khadka et al. 2020; Lei and Berger 2020; Borg et al. 2021). Such lineage-specific histone variants provide exciting opportunities to reveal novel epigenetic requirements and regulatory mechanisms via innovations in histone functions.

All four core RC histone proteins—H2A, H2B, H3, and H4—are present in stoichiometric ratios within nucleosomes. However, there is nonuniform diversification of RC histones into histone variants. For example, there are many H2A variants in mammals but comparatively fewer H3 variants and even fewer H2B and H4 variants (Talbert and Henikoff 2010, 2021). This differential diversification may be due to each histone’s relative position in the nucleosome and its propensity to alter nucleosome properties upon replacement (Malik and Henikoff 2003). Yet, H2B variants have proliferated in other lineages, including plants (Jiang et al. 2020; Török et al. 2016), suggesting that they can be an abundant source of evolutionary and functional diversification. Our recent study revealed previously undescribed H2A variants and their evolutionary origins within mammals (Molaro et al. 2018). Since H2A and H2B histones form obligate heterodimers, we investigated whether there were H2B variants that remain undiscovered in mammalian genomes.

Three H2B variants—H2B.1, H2B.W, and subH2B (referred to as H2B.L, according to HGNC nomenclature)—have been previously described in mammals, where they appear to be specialized for roles in the germline. H2B.1 (also referred to as testis-specific H2B, or TSH2B, or TH2B) was one of the earliest H2B variants to be discovered in mammalian testes (Branson et al. 1975; Shires et al. 1975; Zalensky et al. 2002). This variant is 85% identical in sequence to RC H2B and appears to play a role during spermatogenesis and in postfertilization zygotes (Govin et al. 2007; Montellier et al. 2013; Shinagawa et al. 2014). Structural and in vitro studies suggest that H2B.1-containing nucleosomes are less stable than RC H2B (Li et al. 2005; Urahama et al. 2014), which may allow H2B.1 to facilitate histone-protamine exchange during spermatogenesis. More recently, H2B.1 has also been detected in mouse oocytes, where its function is not yet understood (Montellier et al. 2013; Shinagawa et al. 2014). H2B.W (also referred to as H2BFWT), was detected in sperm and appears to localize to telomeres when expressed in cultured cells (Churikov et al. 2004; Boulard et al. 2006); it remains functionally uncharacterized. SubH2B (for subacrosomal H2B) or H2B.L (Govin et al. 2007), does not appear to function in chromatin, but instead localizes to a perinuclear structure in sperm known as the subacrosome (Aul and Oko 2001). Although this compartment is involved in fertilization, H2B.L’s exact function remains uncharacterized despite its abundant expression in sperm. In addition to these germline H2B variants, a fourth H2B variant, H2B.E, is expressed in olfactory neurons in rodent species. H2B.E differs from RC H2B by only five amino acid residues and plays important roles in regulating neuronal transcription and lifespan (Santoro and Dulac 2012). Preliminary evolutionary analyses of a few previously identified mammalian H2B variants (González-Romero et al. 2010) suggested male germline-enriched variants may have accelerated rates of evolution. However, the evolutionary trajectories of H2B variants in mammals, including their diversity, origins and turnover, and their specialized germline functions are poorly understood.

Here, we perform detailed phylogenomic analyses of mammalian histone H2B variants and describe five evolutionarily distinct H2B variants in mammals, including two novel H2B variants, which we named H2B.K and H2B.N following previously proposed nomenclature guidelines (Talbert et al. 2012). Except for H2B.K, which arose early in vertebrate evolution, all other H2B variants originated in early mammalian evolution and have been largely retained across mammalian orders. Yet, all H2B variants show dramatic expansions and/or pseudogenization, indicative of high evolutionary turnover. Whereas most H2B variants are predominantly expressed in testes or sperm, we find that the newly discovered H2B.K and H2B.N variants are instead overwhelmingly expressed in oocytes and early zygotes. Our analyses also reveal that H2B variants span a vast spectrum of evolutionary rates and have a wide range of sequence divergence from RC-H2B, suggesting that some variants might have evolved unconventional chromatin packaging properties or even nonchromatin functions. Together, our analyses reveal the presence of a larger H2B repertoire in mammals than previously recognized, highlighting the power of evolutionary approaches to uncover innovation of lineage-specific chromatin functions.

## Results

### Seven Distinct H2B Variants in Mammals

**Fig. 1.**
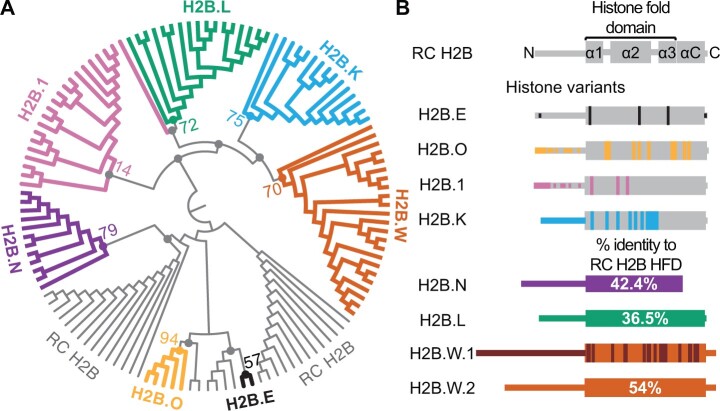
Phylogenomic analyses identify distinct H2B variant clades in mammals. (*A*) A maximum-likelihood protein phylogeny of the HFD of selected ancestral/RC H2B sequences and all intact H2B variants sequences from 18 representative mammalian species is represented as a circular cladogram (see supplementary data 1, Supplementary Material online, for a phylogram with branch lengths scaled to divergence). RC H2B histones are shown in gray, and seven H2B variant clades identified using phylogeny are highlighted in colors: H2B.E (black), H2B.O (yellow), H2B.N (purple), H2B.1 (pink), H2B.L (green), H2B.K (blue), and H2B.W (orange). Bootstrap values at selected nodes with >50% support are shown along with colored dots to indicate the nodes they represent. The H2B.1 clade has a low bootstrap support of 14% owing to its high similarity to RC H2B (see supplementary figs. S1 and S3, Supplementary Material online, for additional information). Select nodes with low bootstrap support values (<20%) are indicated with a gray dot. (*B*) Schematics of RC H2B and H2B variants. A structural schematic of a RC H2B at the top shows the N-terminus, HFD (including the α1, α2, α3 helices, and intervening loops), αC domain, and the C-terminus. Variants with high identity to RC H2B (H2B.E, H2B.O, H2B.1, and H2B.K) are shown in gray with differences from RC H2B colored using the same colors as (*A*). More divergent variants (H2B.N, H2B.L, and H2B.W.1 and H2B.W.2) are represented in solid colors and the percent identities of the HFD and αC domain compared with RC H2B are indicated. Differences between H2B.W.1 and H2B.W.2 are further highlighted in brown to indicate the divergence of these paralogs. Schematics and percent identities are based on human sequences, except for H2B.E and H2B.O, which are only found in some rodents (mouse sequence used) and platypus, respectively, and H2B.L which is pseudogenized in humans, (rhesus macaque sequence used as a reference).

To identify variants of histone H2B in mammals, we interrogated genome assemblies from 18 representative mammals. We performed comprehensive and iterative homology-based searches using both previously identified histone variants and new histone variants identified during our analyses (Molaro and Drinnenberg 2018) (see Materials and Methods). We further determined shared synteny (conserved genomic neighborhood) to identify orthologs. Thus, we were able to obtain a near-comprehensive list of all variant H2B open reading frames (ORFs) in these mammalian genomes. Since RC histones are present in large, nearly identical, multigene arrays, we did not compile all histone sequences that are near-identical to mouse or human RC H2B (Marzluff et al. 2002; Talbert et al. 2012). Although it is possible that some of those gene copies might be RI H2B variants, we focused instead on divergent H2B variants that are clearly distinct from RC H2B. Next, we performed protein sequence alignments of all identified H2B variants to identify incomplete sequences and manually curate our gene annotations. The alignment shows that H2B variants vary considerably in sequence and length in their N-terminal tails, making them difficult to align reliably in this region. Furthermore, we found that the C-terminal αC domain is absent or truncated in a subset of histone variants. Nevertheless, most H2B variants showed higher sequence conservation in their HFD and αC helix than in their tails (fig. 1*B*). To understand the evolutionary relationships between the H2B variant sequences we identified, we performed maximum likelihood phylogenetic analyses using PhyML (Guindon and Gascuel 2003; Guindon et al. 2010). We used only regions we could reliably align across all variants, either an alignment of the HFD and αC domains (fig. 1*A* and supplementary fig. S1, Supplementary Material online) or just the HFD (supplementary fig. S2, Supplementary Material online). We did not observe any substantial differences between phylogenetic groupings or topology in these two analyses.

Our analyses revealed seven distinct clades that represent discrete classes of H2B variants with unique features (fig. 1*A* and supplementary fig. S1, Supplementary Material online). Five of these clades are broadly distributed among mammals, whereas two clades have a restricted species distribution, suggestive of very recent evolutionary origins. The first of these species-restricted clades is *H2B.E*, which was originally identified through functional analyses of olfactory neurons in mice (Santoro and Dulac 2012) (fig. 1). We could only find one unambiguous ortholog of *H2B.E* in the closely related rat genome but none in the more distantly related guinea pig genome (supplementary fig. S1, Supplementary Material online). Since H2B.E differs from most copies of RC H2B by only five amino acid residues (three within the HFD) (Santoro and Dulac 2012), we recognized that phylogenetic analyses alone may not be adequate to identify all *H2B.E* orthologs. Therefore, we turned to shared synteny analyses to search for *H2B.E* orthologs in other mammalian genomes (supplementary fig. S3*A*, Supplementary Material online). Mouse *H2B.E* is found within a small cluster of *RC H2B* genes that is distinct from the major *H2B* cluster (Wang et al. 1996; Marzluff et al. 2002). Aligning all *H2B* genes within the *H2B.E* syntenic locus, we found only a single copy of *H2B* in mouse and rat that shares a majority of the five distinct residues characteristic of the originally identified mouse *H2B.E* (supplementary fig. S3*B*, Supplementary Material online). We extended these analyses to other rodent and lagomorph genomes, revealing strong support for the presence of *H2B.E* orthologs in *Muridae* (supplementary fig. S4*A* and *B*, Supplementary Material online). A key piece of evidence is that these putative orthologs encode proteins that share five amino acid residues, which distinguish H2B.E from RC-H2B (supplementary fig. S4*C*, Supplementary Material online). Based on our analyses, we conclude that *H2B.E* either arose only in *Muridae*, or there are not enough distinguishing sequence features for us to unambiguously identify *H2B.E* orthologs outside *Muridae*. Given this uncertain status, we do not further discuss *H2B.E* in our study.

We also identified a previously undescribed clade of H2B variants that we named *H2B.O* (we follow the histone nomenclature guidelines proposed in Talbert et al. [2012]), which is exclusively found in the platypus genome (supplementary fig. S1, Supplementary Material online). *H2B.O* variants represent a bona fide clade, that is, they group together to the exclusion of all other H2Bs. Their expression appears to be enriched in platypus’ germline tissues (testes or ovaries) albeit at low levels (supplementary fig. S5, Supplementary Material online). Due to their clear absence from the placental mammals, we are unable to draw more significant conclusions about their function and evolutionary constraints.

Of the remaining broadly distributed H2B variants, three (H2B.1, H2B.L, and H2B.W) have been previously described, whereas two (H2B.K, H2B.N) are newly identified by our analysis. Each of the three variants displayed unique evolutionary features. Most of the seven clades have high bootstrap support for the grouping of their orthologs (>50%) to the exclusion of other H2Bs. The only exception was H2B.1 orthologs that grouped together with low confidence (14%), likely due to their very high sequence similarity to RC H2B within the HFD used to generate the phylogeny (fig. 1*B* and supplementary fig. S1, Supplementary Material online). Although the N-terminal tail of most H2B variants is too diverged for use in phylogenetic analysis, H2B.1’s N-terminal tail can be reliably aligned to RC H2B. A phylogeny using a full-length alignment (i.e., including the N-terminal tail) unambiguously distinguished all H2B.1 orthologs from RC H2B with high bootstrap support (100%, supplementary fig. S6, Supplementary Material online). Synteny further supports unambiguous orthology for all the *H2B.1* genes we examined (see below).

The H2B.W clade is also broadly present across mammals (supplementary fig. S1, Supplementary Material online). However, we also found that human *H2B.M*, which is found in genomic proximity to human *H2B.W*, groups within the H2B.W clade (supplementary figs. S1 and S8, Supplementary Material online). Most features that distinguish human H2B.M from human H2B.W lie in the divergent N-terminal tails whereas their HFDs are much more similar (fig. 1*B*). We found many such apparent duplications of *H2B.W* histone variants across mammalian species. Their close proximity to each other could allow copies to recombine or undergo gene conversion, resulting in similar sequences. We performed GARD analyses to test for such signatures of gene conversion and found that mammalian *H2B.W* variants are indeed undergoing recurrent gene conversion with each other, leading to a species-specific clustering pattern (supplementary fig. S1, Supplementary Material online). Based on the established guidelines for histone nomenclature (Talbert et al. 2012), we henceforth refer to this clade as H2B.W in mammals; we refer to human *H2B.W* as *H2B.W.1* and *H2B.M* as *H2B.W.2*.

Like H2B.1 and H2B.W, we found that the H2B.L clade is broadly represented across mammals (supplementary fig. S1, Supplementary Material online) except in humans, where *H2B.L* appears to be a pseudogene. We also found two phylogenetically distinct clades—H2B.K and H2B.N—that have not been previously identified. An unusual feature of both H2B.K and H2B.N is that they are encoded by intron-containing genes, whereas all other H2B variants and RC H2B lack introns (supplementary table S1, Supplementary Material online). The intron in these two variants is in the same location with respect to the HFD, suggesting that *H2B.K* and *H2B.N* may have a common ancestor, although our current phylogeny does not provide adequate support for their common origin.

Thus, our phylogenetic analyses identified seven distinct clades of H2B variants, including five that are broadly distributed among mammals. Although the H2B variant clades are clearly distinct from each other, and well supported by high bootstrap values, we are unable to make any strong inferences about the branching order of the clades, that is, whether they arose from a single duplication from ancestral *RC H2B* and subsequently diversified (monophyletic) or whether they arose via independent duplications of RC H2B (polyphyletic). This poor resolution contrasts with the strong evidence for monophyly (single evolutionary origin) of the short histone H2A variants in mammals (Molaro et al. 2018).

### Structural Features of H2B Variants

**Fig. 2.**
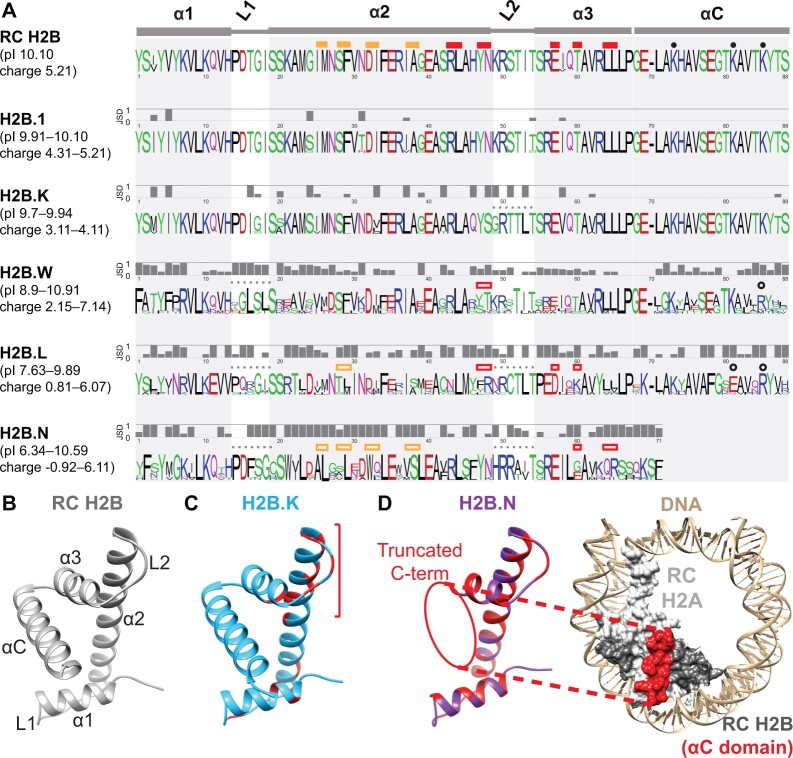
H2B variants diverge from RC H2B and each other in many protein features. (*A*) Logo plots depicting protein alignments of the HFD (α1, L1, α2, L2, α3) and αC domain of RC H2B and H2B variants across an identical set of representative mammals (see Materials and Methods). Colors of residues highlight their biochemical properties: hydrophobic (black), positively charged (blue), negatively charged (red), polar (green), and others (magenta). JSD at each amino acid position were calculated between RC H2B and each H2B variant. Low JSD values indicate low divergence between RC H2B and variant H2Bs, whereas high JSD values indicate that RC H2B and H2B variants have distinct residues. Above the RC H2B logo, we indicate residues that interact with H2A (filled yellow boxes) or H4 (filled red boxes) and residues that are posttranslationally modified (filled circles) (Luger et al. 1997; McGinty and Tan 2021). Changes at these positions in H2B variants are indicated above each logo plot with empty boxes/circles, and loop regions with altered residues are indicated with a dotted line. The ranges of isoelectric points (pI) and charge across orthologs of each variant are shown in parentheses on the left. Note that H2B.N orthologs are missing most of the αC domain. See supplementary figure S7*B*, Supplementary Material online, for Logo plots depicting protein alignments of the N-terminal tail across the same set of representative mammals. (*B*) Structure of the HFD and αC domain of human RC H2B (Arimura et al. 2018). The N and C termini are unstructured. (*C*) Homology model of human H2B.K (blue) indicates a cluster of red sites (bracket) that differ from RC H2B (see Materials and Methods). (*D*) A homology model of human H2B.N (left, purple) with sites that differ from RC H2B highlighted in red (see Materials and Methods). RC H2A-H2B within a nucleosome (PDB:5y0c; Arimura et al. 2018) is shown on the right, with DNA (tan), RC H2A (light gray), and RC H2B (dark gray) surfaces and the RC H2B αC domain (red) highlighted.

To identify key residues that distinguish RC H2B from H2B variants, we compared the HFD and αC of RC H2B with each of the five broadly retained H2B variants. The N-terminal tails showed high divergence and could not be reliably aligned across different variants (supplementary fig. 7*B*, Supplementary Material online). Therefore, we decided to focus on the HFD and αC domain to make any reliable inferences. We chose orthologs from seven mammals, all of which encode at least one intact copy of each H2B variant (fig. 2*A*). We aligned orthologs of RC H2B and H2B variants and created logo plots to visualize their sequence conservation (see Materials and Methods). To investigate each variant’s divergence from RC H2B, we calculated the Jensen–Shannon distance (JSD) at each position, comparing a set of seven orthologs of each variant with a set of seven orthologs of RC H2B from the same species. High JSD values indicate between-paralog differences that are also conserved within both groups of orthologs. We did not identify any residues that are conserved across all H2B variants, but different from RC H2B. However, we identified residues that are conserved across orthologs of each H2B variant but distinct from RC H2B (high JSD, >0.75, fig. 2*A*). We mapped these variant-specific conserved residues onto homology models constructed using a previously described structure of human RC H2B (PDB:5y0c, fig. 2*B*) (Arimura et al. 2018).

We find that H2B.1 and H2B.K are highly similar to RC H2B in their HFD (figs. 1*B* and 2*A*). H2B.1 differs from RC H2B by only three conserved differences in the HFD (JSD ∼1.0) (fig. 2*A* and supplementary fig. 7*A*, Supplementary Material online). The N-terminal tail of H2B.1 differs more from RC H2B (supplementary fig. 7*B*, Supplementary Material online), including at S/T residues that can be phosphorylated in RC H2B (Zalensky et al. 2002; Li et al. 2005). With this exception, all residues important for H2A (yellow bars), H4 (red bars) or DNA interaction and PTM residues (black dots) are identical to RC H2B, suggesting that H2B.1 and RC H2B share similar protein and chromatin properties.

Similarly, H2B.K orthologs have only a few fixed differences from RC H2B within the H2A-, H4-interacting residues, and PTM sites, suggesting that these properties are likely conserved between RC H2B and H2B.K. Instead, most of the changes that distinguish H2B.K from RC H2B occur in other HFD sites, with several clustered around the second DNA binding loop (L2, fig. 2*A* and *C*) that could affect DNA binding or specificity. Furthermore, H2B.K is predicted to have a slightly lower charge than RC H2B (fig. 2*A*) that might result in less tightly packed DNA. In contrast to its HFD, H2B.K’s N-terminal tail differs dramatically from RC H2B (supplementary fig. 7*B*, Supplementary Material online). For example, H2B.K’s N-terminal tail is missing key lysine residues that are posttranslationally modified in RC H2B. Atypically for H2B proteins, H2B.K also has a variable-length polyglutamine tract in its N-terminal tail that could facilitate protein–protein interactions (Schaefer et al. 2012). Since H2B.K is a newly identified histone variant, its biochemical properties remain uncharacterized.

The remaining H2B variants (H2B.W, H2B.L, H2B.N) share less than 50% amino acid identity with RC H2B in their HFD. For example, many residues in the HFD of H2B.W are conserved among orthologs, but diverged from RC H2B (JSD∼1, fig. 2*A*); these appear to cluster in the homology model (supplementary fig. 7*A*, Supplementary Material online). In spite of these differences, most H2A- and H4-interacting residues and PTM sites are conserved between H2B.W and RC H2B (fig. 2*A*). Divergence is even greater in their N-terminal tails, which cannot be reliably aligned with RC H2B (supplementary fig. 7*B*, Supplementary Material online). Human H2B.W.1 and H2B.W.2 also diverge most from each other in their N-terminal tail (fig. 1*B*). Unlike all other H2B variants, H2B.W variants have an extended C-terminal tail in some mammals (including humans), which is nearly identical between H2B.W.1 and H2B.W.2 in primates. Their divergence results in an unusually wide range of charge and isoelectric points within H2B.W variants.

Even though H2B.L localizes to the subacrosome in sperm (Aul and Oko 2001), it is nonetheless capable of localizing to chromatin in the nucleus when expressed in cell lines (Tran et al. 2012). Putative H4-interacting residues, L2 residues, and PTM residues are different between H2B.L and RC H2B (open red/yellow boxes, fig. 2*A* and supplementary fig. 7*A* and *B*, Supplementary Material online). These differences may contribute to its unusual biological role outside the nucleus.

Finally, H2B.N shows the most dramatic differences from RC H2B in the HFD (fig. 2*A*). Although H2A-, H4-interacting residues, and residues in L2 are largely conserved between H2B.N orthologs, they are highly divergent from RC H2B. The most striking difference is that most H2B.N orthologs are significantly truncated in their C-terminus. Homology modeling predicts that this truncation results in the loss of the αC domain, whose residues are part of the essential nucleosome acidic patch (Nacev et al. 2019; McGinty and Tan 2021) (fig. 2*D*). This suggests that the unusual H2B.N could endow nucleosomes with unique properties, or that H2B.N might have evolved nonnucleosomal functions, like H2B.L.

### Evolutionary Origins of Mammalian H2B Variants

**Fig. 3.**
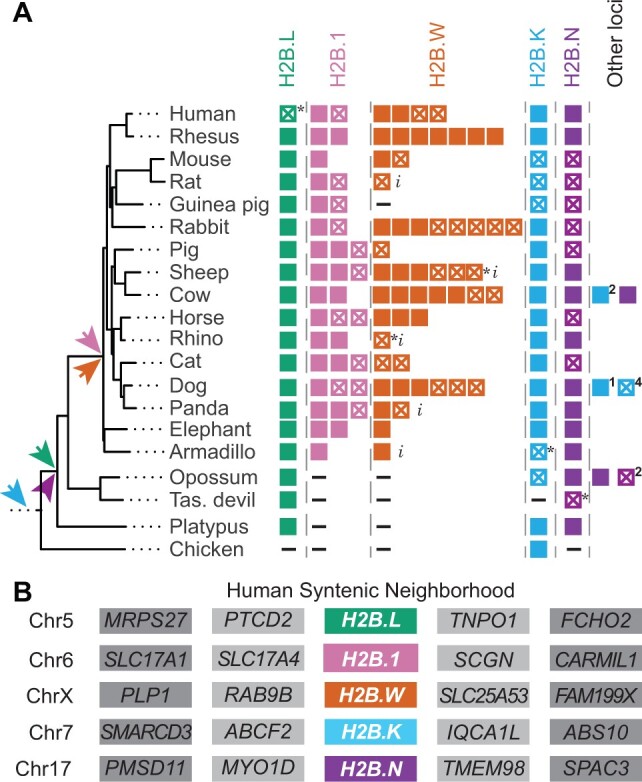
Retention and synteny of identified H2B variants in mammals. (*A*) A schematic representation of H2B variants and paralogs, along with a species tree of selected representative mammals and a nonmammalian outgroup, chicken, (Bininda-Emonds et al. 2007). Colors distinguish H2B variants as in figure 1*A*. Filled boxes represent intact ORFs and empty boxes with a cross represent interrupted ORFs (inferred pseudogenes). An asterisk (*) indicates pseudogenization by a single-nucleotide change which could either be sequencing error or a true mutation. A dash (–) indicates absence of histone within the syntenic location, and an “*i*” indicates incomplete sequence information. Colored arrows indicate the predicted origin of each H2B variant. Copies of variants found outside the syntenic neighborhood are shown as “other loci” with number of intact ORFs and pseudogenes indicated. (*B*) A schematic of the shared syntenic genomic neighborhoods of each H2B variant in the human genome. All H2B variants in (*A*) were present in the same syntenic location across mammals (see supplementary fig. S8, Supplementary Material online, for detailed syntenic analyses) except “other loci.”

To identify the age and subsequent evolutionary patterns of the five mammal-wide clades of H2B variants, we searched genome assemblies of representative mammals and an outgroup, chicken. We classified uninterrupted ORFs as intact genes (fig. 3*A*). We made the distinction between pseudogenes with many frame-disrupting mutations versus those that are only a single point mutation away from encoding an intact ORF (indicated with an asterisk); the latter could represent sequencing errors in otherwise intact ORFs. We found that all H2B variants have been largely retained across mammals at their shared syntenic location (fig. 3*B* and supplementary fig. S8, Supplementary Material online). Based on their presence in all eutherian (placental) mammals, we infer that both *H2B.1* and *H2B.W* clades arose in the last common ancestor of eutherian mammals (∼105 Ma), whereas the *H2B.L* and *H2B.N* clades also contain marsupial and platypus (but not chicken) sequences, and therefore arose in the last common ancestor of all mammals (∼177 Ma) (fig. 3*A*) (divergence times calculated using TimeTree estimates; Hedges et al. 2015).

*H2B.K* is the only H2B variant for which we could identify an ortholog in the shared syntenic location in chicken, a nonmammalian outgroup (fig. 3*A*). We extended our analyses to other vertebrates and found *H2B.K* orthologs at least as far back as bony fishes in shared syntenic locations (supplementary fig. S9*A* and *B*, Supplementary Material online). *H2B.K* orthologs from vertebrates also group with the previously identified “cleavage-stage dependent” histones in sea urchin (Kemler and Busslinger 1986; Lai et al. 1986; Marzluff et al. 2006). Cleavage-stage histones, first described in sea urchin, are expressed at specific stages in embryogenesis. Although orthologs of these sea urchin histones have been identified in other vertebrates (Ohsumi and Katagiri 1991; Mandl et al. 1997; Tanaka et al. 2001), our phylogeny lacks the bootstrap support for us to assign H2B.K and sea urchin cleavage-stage H2Bs to the same clade. The fragmented nature of the sea urchin genome assembly does not allow us to use shared synteny analysis to increase our confidence in assigning these to the same clade. Some sea urchin H2Bs show an extended N-terminal tail with a pentapeptide repeat sequence (Brandt and von Holt 1978; Strickland et al. 1978). However, within the vertebrate H2B.K N-terminal tail, we were unable to find any obvious sequence similarity or an extended repeat sequence as in sea urchin H2Bs (supplementary fig. S9*C*, Supplementary Material online). Finally, the presence of an intron in all *H2B.K* orthologs (which is also present in *H2B.N* orthologs) but not in sea urchin cleavage-stage H2Bs challenges their orthology. Overall, our analyses suggests that four H2B variants arose in mammals, whereas *H2B.K* likely originated in the common ancestor of bony vertebrates (∼435 Ma), although this may even be an underestimate of its age.

### Rapid Gene Turnover of H2B Orthologs

Next, we investigated the evolutionary dynamics of each H2B variant after its birth. We examined duplications, losses, and rates of protein sequence change. We found that all H2B variants, except *H2B.L*, have experienced additional lineage-specific gene duplications (fig. 3*A*). *H2B.1* and *H2B.W* duplications occurred near or within the original syntenic locus in multiple mammals (fig. 3*A* and supplementary fig. S8, Supplementary Material online). In contrast, we found intronless duplicates of *H2B.K* and *H2B.N* in nonsyntenic locations (other loci in fig. 3*A*), suggesting they arose via retrotransposition of their intron-containing progenitor genes. Notably, this pattern of gene duplication and retroposition often appears lineage-specific, with paralogs grouping with intron-bearing genes from the same species, suggesting this retroposition occurred more recently (Yang et al. 2020). Based on this, we infer that *H2B.K* and *H2B.N* are likely to be expressed in the germline, since that is the only tissue in which retrogenes can be heritably integrated into the genome (supplementary fig. S1, Supplementary Material online).

**Fig. 4.**
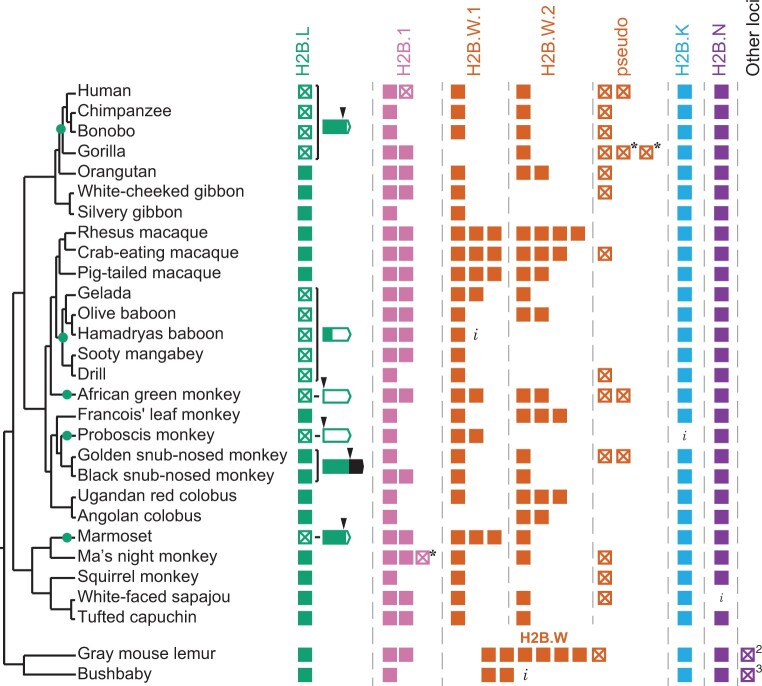
Evolutionary dynamics of H2B variants in primates. Schematic representation of identified H2B variants in primates, shown to the right of a species tree of primates (Perelman et al. 2011; Osada 2015; Wright et al. 2015). Colors distinguish H2B variants as in figure 1*A*. Filled boxes represent intact ORFs and empty boxes with a cross represent inferred pseudogenes. An asterisk (*) indicates pseudogenization by a single-nucleotide change which could either be sequencing error or a true mutation. An “*i*” indicates incomplete sequence information. Copies of variants found outside the syntenic neighborhoods are shown as “other loci” with number of copies indicated. Green dots on the tree represent H2B.L pseudogenization events inferred based on shared pseudogenizing mutations between species. For H2B.L copies with pseudogenizing mutations or mutations that dramatically alter the ORF, disruptions to the ORF are detailed on a gene schematic. Black arrowheads indicate nucleotide changes that result in a stop codon, frameshift, or loss of start codon. Intact sequences in the ORF are filled green in the gene structure and disrupted sequences are empty. Black box indicates extension of ORF in snub-nosed monkeys. See supplementary figure S10, Supplementary Material online, for more detailed description of H2B.L mutations. H2B.W.1 and H2B.W.2 can be distinguished in simian primates using phylogeny (see supplementary fig. S12, Supplementary Material online) and so are shown in separate columns.

Except for *H2B.1*, we found that no other H2B variant is universally retained in all mammals; each is pseudogenized in at least one mammalian species (fig. 3*A*). For example, both *H2B.K* and *H2B.N* were pseudogenized in rodents. Our initial survey revealed that the human genome appears to encode a *H2B.L* pseudogene, which is a single mutation away from encoding an intact ORF. Given the rarity of pseudogenization among mammalian *H2B.L* genes, we investigated *H2B.L* more closely across primates. We found that the frameshifting mutation (and subsequent early stop codon) found in humans is also present in chimpanzee, bonobo, and gorilla, suggesting that a true pseudogenization event occurred ∼9 Ma in *Homininae* (figs. 3*A* and 4; supplementary fig. S10, Supplementary Material online). We also found that *H2B.L* pseudogenized at least five independent times in simian primates (fig. 4 and supplementary fig. S10, Supplementary Material online). Thus, unusually among mammals, the subacrosomal H2B.L variant appears to be nonfunctional in many primates.

In contrast to *H2B.L*, humans and other primates encode at least one intact copy of other H2B variants (fig. 4). Like most mammals, *H2B.K* and *H2B.N* are present in single copy in all primates, whereas *H2B.1* and *H2B.W* are present in multiple copies. Many primates have two copies of *H2B.1* that diverged from each other in the last common ancestor of simian primates (supplementary fig. S11, Supplementary Material online), although some species (including humans) subsequently lost one paralog. As we observed in our broader sample of mammals, *H2B.W* experienced dramatic duplications and pseudogenization in primates (fig. 4). However, unlike in other mammals, simian primate *H2B.W.1* and *H2B.W.2* genes can be readily distinguished by phylogenetic analyses of their HFDs. This suggests that primate *H2B.W.1* and *H2B.W.2* no longer experience gene conversion in their HFD and might have acquired partially nonredundant functions in primates. However, *H2B.W* gene turnover appears to be still active in primates; some primates have additional copies of H2B.W that do not reliably group with either H2B.W.1 or H2B.W.2, whereas other primates are missing an intact copy of either *H2B.W.1* or *H2B.W.2* (supplementary fig. S12, Supplementary Material online).

H2A and H2B form heterodimers before being incorporated into nucleosomes, suggesting that they might co-evolve. Previous work has identified dramatic diversification of H2A variants, especially short H2A variants in mammals (Govin et al. 2007; Ferguson et al. 2009; Shaytan et al. 2015; Draizen et al. 2016; Molaro et al. 2018). However, with one exception, we did not observe any obvious correlations between the evolution of H2B and H2A variants when we examined their shared presence/absence in mammals. The one exception is that *H2A.1* and *H2B.1* are found in the same locus and share regulatory elements (Huh et al. 1991). We found that a duplication of *H2B.1* is often accompanied by a duplication of *H2A.1* (supplementary fig. S8, Supplementary Material online). However, pseudogenization of one variant does not always lead to pseudogenization of the other. Thus, we cannot distinguish whether the apparent coevolution of *H2A.1* and *H2B.1* is due to genomic proximity and/or functional selection.

Overall, our phylogenomic studies of mammalian H2B variants reveal a dramatic, recurrent pattern of gene duplication and occasional functional loss. Lineage-specific loss of some H2B variants suggests that they are not essential for viability or fertility. Alternatively, the H2B variants might collectively perform an essential function but are individually functionally redundant.

### Evolutionary Diversification and Selective Constraints Acting on H2B Variants

**Fig. 5.**
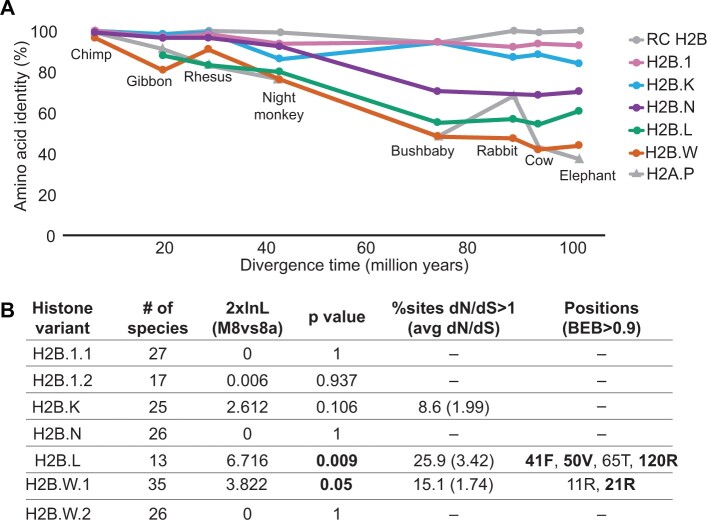
Evolutionary tempo of H2B variants in mammals. (*A*) Pairwise amino acid identity of H2Bs and H2A.P (Molaro et al. 2018) showing comparisons of specified mammal orthologs versus either the human or orangutan (for H2B.L) ortholog of each H2B variant. Percent identities (*y* axis) are plotted against species divergence time (*x* axis). For H2B variants with multiple copies, the copy with the highest identity to the human ortholog was used (see supplementary table S2, Supplementary Material online; Materials and Methods) to be conservative. (*B*) PAML analyses were used to look for site-specific positive selection (supplementary table S4, Supplementary Material online). Log likelihood differences and *P* values from the Model 8 versus Model 8a comparison are indicated. For the variants where likelihood tests suggest the presence of positive selection, the percentage of sites with d*N*/d*S*>1 is shown, along with the estimated average d*N*/d*S* for those sites. The “Positions” column lists positively selected sites (M8 BEB>0.9); sites also identified by FUBAR analyses are highlighted in boldface (Murrell et al. 2013). Amino acid residues shown correspond to the rhesus macaque protein sequence. See supplementary figure S13, Supplementary Material online, for alignments showing positively selected residues.

Given the long branch lengths of some H2B variants in our phylogeny (supplementary figs. S1 and S2, Supplementary Material online) and the diversity revealed in their HFDs (fig. 2*A*), we hypothesized that some H2B variants may have evolved more rapidly than RC H2B. To investigate this possibility, we compared the rate of protein divergence of RC H2B and H2B variants in a representative group of mammals spanning 100 My of evolution (fig. 5*A* and supplementary table S2, Supplementary Material online). For comparison, we also included the *H2A.P* variant, which is one of the most rapidly diverging histone variants in mammals (Molaro et al. 2018). We measured the pairwise identity of each mammalian H2B protein to its human ortholog (or orangutan ortholog for *H2B.L*, since human *H2B.L* is a pseudogene) and plotted it as a function of species divergence time (using TimeTree estimates; Hedges et al. 2015). To be conservative, we chose the least divergent ortholog when multiple paralogs were found in the shared syntenic location. As expected, the highly conserved RC H2B shows the slowest rate of protein divergence. H2B.1 and H2B.K also evolve slowly. In contrast, H2B.N and H2B.L exhibit an intermediate rate, whereas H2B.W shows the fastest rate of protein divergence among H2B variants, comparable with a rapidly evolving H2A variant, H2A.P (fig. 5*A*).

The faster rates of amino acid change we observe could indicate diversifying (positive) selection at a select number of sites, or relaxed constraint. For example, complete lack of constraint would imply no functional selection for protein-coding capacity (i.e., neutrally evolving pseudogenes). To test for neutral evolution, we evaluated H2B variants by examining the ratio of rates of nonsynonymous (amino acid altering, d*N*) to synonymous (d*S*) changes. Neutrally evolving sequences have d*N*/d*S* ratios close to 1, whereas strong purifying selection results in ratios near 0, with most nonsynonymous changes disallowed. Using the same set of species selected above (fig. 5*A*) as input for PAML analysis (Yang 1997), we compared the relative likelihoods of models that assume sequences evolve neutrally versus those that allow nonneutral evolution. Specifically, we used PAML’s codeml program to estimate the likelihood of a simple evolutionary model (Model 0), where all sequences and all codons are assumed to have the same d*N*/d*S* ratio. We compared the likelihood of model 0 with d*N*/d*S* fixed at 1 (neutral) with that of model 0 with d*N*/d*S* estimated from the alignment. Using this test, we found strong evidence for purifying selection for all H2B variants across mammals (supplementary table S3, Supplementary Material online), rejecting neutrality and the possibility that their high protein divergence is due to pervasive pseudogenization. Furthermore, the overall d*N*/d*S* estimates from these analyses are consistent with the relative evolutionary rates of the H2B variants based on protein divergence (fig. 5*A*).

The signatures of overall purifying selection in the H2B variants do not rule out the possibility that a subset of sites might nevertheless evolve under positive selection (d*N*/d*S* > 1). Indeed, H2B variants in plants (Jiang et al. 2020) and short H2A variants in mammals (Molaro et al. 2018) show evidence of both overall purifying selection and positive selection at selected sites. To investigate this possibility, we analyzed H2B variant sequences from simian primates, a clade with a level of evolutionary divergence that is ideal for codon-by-codon analyses. We analyzed intact ORF sequences from 27 species for most variants except for *H2B.L* (13 species) due to its recurrent pseudogenization in many primates (fig. 4 and supplementary fig. S10, Supplementary Material online). We performed maximum likelihood analyses using PAML (Yang 1997) and FUBAR (HyPhy package; Pond et al. 2005; Murrell et al. 2015) to investigate whether a subset of codons experience positive selection. Using PAML, we identified signatures of positive selection for *H2B.L* and *H2B.W*, but not for the other variants. We found a strong signature of diversifying selection for *H2B.L*, with an estimated 25.9% of sites evolving with an average d*N*/d*S* of 3.42; four sites in the HFD showed high posterior probabilities of evolving under positive selection (fig. 5*B* and supplementary table S4 and fig. S13, Supplementary Material online). An analysis of 35 *H2B.W.1* paralogs/orthologs from simian primates also revealed diversifying selection, with an estimated 15.1% of sites evolving with an average d*N*/d*S* of 1.74 (fig. 4*B* and supplementary table S4, Supplementary Material online). However, only one site in the H2B.W.1 N-terminal tail showed a high posterior probability of positive selection (fig. 5*B* and supplementary fig. S13, Supplementary Material online). FUBAR analyses also identified additional sites in H2B.L and H2B.W that might have undergone positive selection (posterior probability > 0.9) (supplementary table S4, Supplementary Material online). Unlike PAML analyses, FUBAR analyses also identified *H2B.W.2, H2B.1, H2B.K*, and *H2B.N* as having undergone diversifying selection in simian primates, along with *H2B.L* and *H2B.W* (supplementary table S4, Supplementary Material online). Our limited understanding of functional residues in H2B variants prevents us from making any informative biological predictions about the rapidly evolving sites. Overall, our findings of strong purifying selection suggests that H2B variants perform vital functions leading to their overall retention, whereas our findings of positive selection suggest that they have been subject to recurrent genetic innovation.

### H2B Variants Are Expressed in Mammalian Germlines

Most H2B variants in this study remain functionally uncharacterized. To begin to explore their function, we examined their expression in mammals. Similar analyses had previously revealed putative germline-specific functions of many rapidly evolving H2A variants (Molaro et al. 2018, 2020). Prior studies have shown that some H2B variants are primarily expressed in testes of rodents, bull, or human. For others, including the novel variants identified in this study, the site of expression is not known.

We examined publicly available RNA-seq data for the expression of all H2B variants in diverse somatic (brain, liver, kidney, heart) and germline (testes and ovaries) tissues from a wide range of species—opossum, dog, pig, mouse, human, and chicken (supplementary table S5, Supplementary Material online; see Materials and Methods). For comparison, we included a previously published “housekeeping” gene (*C1orf43*) that is ubiquitously expressed (Eisenberg and Levanon 2013). We did not detect expression of any H2B variants in somatic tissues or most embryonic stem cell lines but we did detect robust expression in the majority of germline samples (supplementary fig. S14, Supplementary Material online).

The H2B.L protein was originally isolated from bull and rodent sperm (Aul and Oko 2001). In our RNA-seq analysis, we found that *H2B.L* is abundantly expressed in testes of representative mammals, except humans, where it is pseudogenized (supplementary fig. S14*A*–*G*, Supplementary Material online). Although the pseudogenizing mutation in humans occurred relatively recently (fig. 4), absence of detectable *H2B.L* implies either that its regulatory sequences are also nonfunctional or that its transcript is rapidly degraded in human cells. In contrast, we observed low levels of *H2B.L* transcript in the testes of rhesus macaque, where the ORF is intact (supplementary fig. S14*I*, Supplementary Material online).

Consistent with previous work in mice (Branson et al. 1975; Shires et al. 1975; Zalensky et al. 2002; Govin et al. 2007; Montellier et al. 2013), we found that *H2B.1* is expressed in testes and ovaries of all mammals we analyzed (supplementary fig. S14*B*–*G*, Supplementary Material online). Among multiple mouse and human embryonic stem cells we analyzed, we only detected *H2B.1* expression in one mouse embryonic stem cell data set (supplementary fig. S14*D* and *H*, Supplementary Material online) implying that *H2B.1* may be expressed at very low levels in embryonic stem cells.

We found that both *H2B.N* and *H2B.K* are expressed in ovaries of opossum, dog, and humans, whereas *H2B.K* is expressed in both testes and ovaries in pigs (supplementary fig. S14*A*–*G*, Supplementary Material online). We did not examine the expression of either *H2B.N* and *H2B.K* in mice, or of *H2B.N* in pigs, because these genes have multiple pseudogenizing mutations in these species (supplementary fig. S14*C* and *D*, Supplementary Material online). We also found *H2B.K* expression in chicken ovaries (supplementary fig. S14*J*, Supplementary Material online), demonstrating that ovarian expression of *H2B.K* likely predates the divergence of birds and mammals.

Previous studies had reported *H2B.W.1* expression in human sperm (Churikov et al. 2004; Boulard et al. 2006). Expression of human H2B.W.1 and H2B.W.2 protein is also enriched in sperm samples in a publicly available expression database (Human Protein Atlas) (Uhlén et al. 2015; Thul et al. 2017; Uhlen et al. 2017) further supporting their expression in the male germline. In contrast to these previous studies, we detected no or very low levels of *H2B.W* expression in all species we examined (supplementary fig. S14*B*–*G*, Supplementary Material online).

We were concerned about the inconsistency between our and previous analyses about the expression of some H2B variants (especially *H2B.W*). We speculated that this inconsistency might be due to unusual RNA structures or tissue heterogeneity. Instead of poly-A tails, RC histone transcripts have unusual stem-loop RNA structures at their 3′ ends that bind stem-loop binding protein, which regulates their stability and translation (Dávila López and Samuelsson 2008; Marzluff et al. 2008). Because of this, RC histones are typically underrepresented in poly(A)-selected RNA-seq data sets. In contrast to RC histones, most histone variants are thought to have polyadenylated transcripts and lack stem loops. Yet, previous work has suggested that RC histones and a histone variant, H2A.X can have alternate mRNA processing modes (Molden et al. 2015; Griesbach et al. 2021). To investigate this dichotomy in RNA structure further, we searched for stem loop structures and poly(A) signals close to the stop codons of all H2B variant genes. Stem loop sequences are easily recognized, whereas poly(A) signal detection is less accurate, with false-positive and false-negative findings. We were able to detect a poly(A) signal in the 3′-UTR of most *H2B.L*, *H2B.N*, and *H2B.K* genes (supplementary fig. S15, Supplementary Material online). Unexpectedly, we detected both stem-loop and poly(A) sequences in the 3′-UTRs of the *H2B.1* and *H2B.W* genes (supplementary fig. S15, Supplementary Material online). Our analyses further reveal that histone variants may also be subject to alternate processing; this layer of histone processing and regulation has been poorly studied. We speculate that alternate RNA processing for some H2B variant genes might have affected our ability to detect *H2B.W.1* and *H2B.W.2* transcripts in publicly available RNA-seq data sets that mostly use poly(A) selection.

**Fig. 6.**
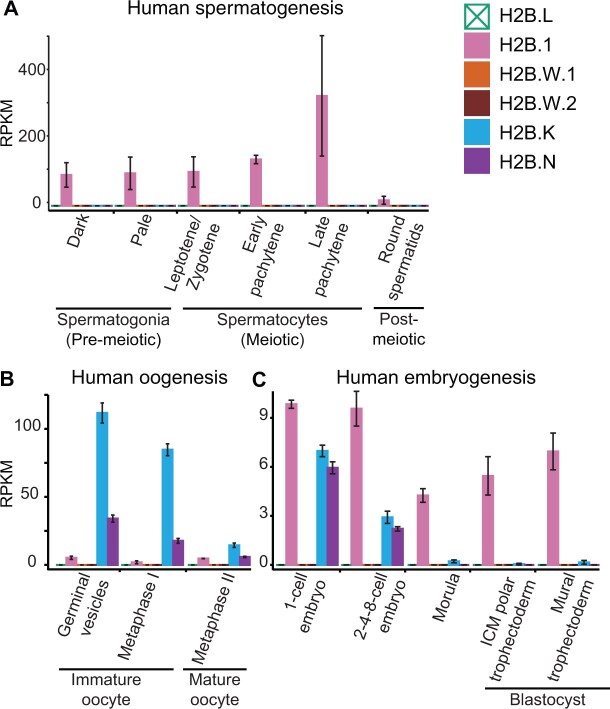
H2B variant expression during human gametogenesis and embryogenesis. RNA expression of H2B variants (reads per kilobase per million mapped reads, RPKM) in publicly available bulk RNA-seq data across different stages of (*A*) human spermatogenesis, (*B*) human oogenesis, and (*C*) human embryogenesis. Legend shows colors corresponding to each histone variant (note that H2B.L is pseudogenized in humans). The bar heights show median RPKMs of biological replicates and error bars show median absolute deviations. See supplementary figure S14, Supplementary Material online, for additional analyses of H2B variants’ expression in somatic and reproductive tissues of other mammals.

A second challenge for detecting histone variant expression in RNA-seq analyses could be cell heterogeneity. For example, many different cell types and developmental stages are present in testes and ovaries. Bulk RNA-seq analyses may be unable to detect robust expression if H2B variants are only transcribed in a small subset of cells. To more closely investigate this possibility, we examined expression of H2B variants during human spermatogenesis. We detected robust expression of *H2B.1* in sperm, with expression increasing during early stages of spermatogenesis (fig. 6*A* and supplementary fig. S14*F*, Supplementary Material online) but decreasing postmeiosis, consistent with previous reports (van Roijen et al. 1998; Govin et al. 2007; Montellier et al. 2013). In contrast, we did not detect expression of *H2B.W.1* or *H2B.W.2* in either spermatogenesis or oogenesis data sets (fig. 6*A* and *B*; supplementary fig. S14*F* and *G*, Supplementary Material online). It is possible that both *H2B.W* variants are expressed at stages of gametogenesis (Churikov et al. 2004) that are not captured in our data analyses due to lack of poly(A) tails at the 3′ end of their transcripts (above). Alternatively, even low expression of *H2B.W* variants may be sufficient for their function in sperm.

Analyses of human oogenesis revealed robust expression of *H2B.K* and *H2B.N* in oocytes, with levels increasing across oogenesis (fig. 6*A* and *B*; supplementary fig. S14*F* and *G*, Supplementary Material online). Neither *H2B.K* nor *H2B.N* were detected in granulosa cells, which are the somatic cells of the female germline, suggesting again that expression is restricted to the germline. We also detected low expression of *H2B.1* in human oogenesis (fig. 6*B*), consistent with previous analyses of mouse oogenesis (Montellier et al. 2013; Beedle et al. 2019). Finally, we detected expression of *H2B.1* (consistent with a previous study in mice; Montellier et al. 2013), *H2B.K*, and *H2B.N*, but not of any of the other H2B variants during embryogenesis (fig. 6*C* and supplementary fig. S14*G*, Supplementary Material online). Overall, our analyses suggest that expression of most H2B variants is restricted to the male germline. However, newly identified histones *H2B.N* and *H2B.K* are primarily expressed in ovaries and early embryos, where they may play key roles in female fertility and early development like the cleavage stage histones of sea urchins (Poccia et al. 1981; Tanaka et al. 2001; Oliver et al. 2003).

## Discussion

Histones perform the critical task of packaging genomes and regulating important DNA-based processes (e.g., transcription, DNA repair, chromosome segregation) in most eukaryotes. Because of their critical genome-wide functions, many RC histones evolve under extreme evolutionary constraint, permitting only limited changes in protein sequence even over nearly a billion years of divergence between protists and humans. In contrast, histone variants can acquire changes that enable them to elaborate new functions, contributing to the eukaryotic nucleosome’s remarkable structural and functional plasticity. Understanding the evolutionary history of histones can reveal how biological challenges faced by different organisms have been resolved by chromatin innovation.

We reveal an extensive repertoire of H2B variants in mammalian lineages, including three previously undescribed histone variants (*H2B.O, H2B.K, H2B.N*). Two H2B variants are only found in a small subset of mammals—*H2B.E* in *Muridae* and *H2B.O* in platypus—whereas five variants (H2B*.L, H2B.1, H2B.N, H2B.K, H2B.W*) are found more extensively across eutherian mammals. We find that one of the newly discovered variants, *H2B.K*, arose prior to the origin of bony vertebrates. Given its age, slow divergence, and widespread retention, it is somewhat surprising that *H2B.K* has escaped detection until now. We attribute this to the difficulty of correctly classifying histone variants when interrogating single genomes (e.g., human or mouse). The atypical absence of *H2B.K (*and *H2B.N*) from the mouse genome, where the most extensive characterization of variant histones has been carried out, likely exacerbated this difficulty (Aul and Oko 2001; Govin et al. 2007; Montellier et al. 2013; Shinagawa et al. 2015). Both these reasons further highlight the value of comprehensive phylogenomic studies in identifying and classifying meaningful functional innovation in histone variant genes.

H2B variants display a range of evolutionary divergence rates across mammals. *H2B.1* and *H2B.K* evolve slowly, whereas *H2B.L, H2B.N*, and *H2B.W* evolve more rapidly. We also detected signatures of positive selection for a subset of residues in several H2B variants in simian primates. These observations are consistent with previous work that suggests male germline-specific genes tend to evolve more rapidly (Retief and Dixon 1993; Wyckoff et al. 2000; Torgerson et al. 2002; Turner et al. 2008; Martin-Coello et al. 2009). In addition to divergence in their HFD, H2B variants show significant divergence from RC H2B in their N- and C-terminal tails, so much so that many H2B variant tails cannot be reliably aligned with RC H2B. The most dramatic changes were seen for H2B.W variants, which have significantly longer N- and C-terminal tails, and H2B.N variants, which have much shorter C-terminal tails; these changes could significantly impact nucleosome packaging and stability. Furthermore, these differences in the tail could contribute to altered PTMs on the tails that are crucial to chromatin interactions and their regulation. Even the relatively conserved H2B.1 variant differs from RC H2B at some sites that can be posttranslationally modified (Zalensky et al. 2002; Li et al. 2005; Lu et al. 2009) to facilitate looser packaging of chromatin than by RC H2B (Rao and Rao 1987; Singleton et al. 2007; Urahama et al. 2014). However, none of the H2B N-terminal tails described in our work resembled the extended H2B N-terminal tails with pentapeptide repeats previously described in sea urchin (Brandt and von Holt 1978; Strickland et al. 1978). Thus, the newly identified H2B variants represent a rich source of additional structural, functional, and regulatory complexity.

All variants except *H2B.1* have been lost in at least one mammalian genome. This suggests that all H2B variants except *H2B.1* might be dispensable for viability and/or fertility. Moreover, even loss of *H2B.1* in knockout mice can be compensated for by RC H2B with unusual PTMs that allow for similar nucleosome structure as H2B.1 (Montellier et al. 2013). Yet, double mutant mice of *H2B.1* and *H2A.1* are infertile and inviable (Shinagawa et al. 2015). One explanation for this inviability is that although RC H2B can compensate for H2B.1 function, RC H2A does not appear to compensate for H2A.1, leading to a stoichiometric imbalance (Shinagawa et al. 2015). Since the properties of variant nucleosomes can be affected by variation in any of the four histone components, this represents an additional layer of chromatin complexity and innovation that remains almost entirely unexplored.

Whereas *H2B.E* is expressed in neurons (Santoro and Dulac 2012), all other mammalian H2B variants have germline-biased expression. *H2B.L, H2B.1, H2B.W*, and *H2B.O* are expressed in testis/sperm, whereas *H2B.1, H2B.N*, and *H2B.K* are expressed in ovaries/oocytes and embryos. Together with the invention of germline-enriched short H2A variants in mammals (Molaro et al. 2018), our study reiterates the important role of evolutionary innovation of chromatin functions in mammalian germ cells, similar to what has been previously observed in plants (Jiang et al. 2020). Spermatogenesis may require constant chromatin innovation since it is a hotbed of genetic conflicts (Moore and Haig 1991; Moore and Reik 1996; Torgerson et al. 2002; Civetta and Ranz 2019), both within genomes (e.g., transposable elements, postmeiotic segregation distortion) and between genomes (e.g., sperm competition during fertilization). Given its subacrosomal localization, *H2B.L* is more likely to play a role in gamete fusion rather than in chromatin. However, *H2B.L* appears to be nonfunctional in most simian primates and its function remains uncharacterized in any mammal.

Spermatogenesis in mammals and many other animal species also involves the near-complete replacement of histones by highly charged basic proteins, called protamines, which ensure tight DNA packaging in sperm heads (Oliva and Dixon 1991; Hammoud et al. 2009). Following fertilization, the paternal genome must be stripped of protamines and repackaged by histones, such that paternal and maternal genomes (which never undergo protamine replacement) can initiate embryonic cell cycles in an orderly fashion. Deposition of H2B.1 is a key transitional step between RC histone and protamine-packaged genomes in early spermatocytes (Montellier et al. 2013; Shinagawa et al. 2015) and for repackaging the protamine-rich paternal genome into histones following fertilization (Montellier et al. 2013). Notably, even after the protamine transition, 10–15% of basic nuclear proteins in mature sperm still constitute histones (Tanphaichitr et al. 1978; Rousseaux et al. 2005). Even though H2B.1 is removed from mature sperm, other functionally uncharacterized H2B variants may be required for as-yet-unknown critical functions for spermatogenesis. For example, ectopically expressed H2B.W.1 appears to localize to telomeric chromatin in cell lines (Churikov et al. 2004), suggesting it might play a “bookmarking” role, as has been hypothesized for histones postfertilization (Hammoud et al. 2009). Although several H2B variants appear to be highly enriched in the male germline, this does not eliminate the possibility that they might play important roles during oogenesis and early embryogenesis. For example, the H2A.B variant is testis-enriched in expression but nevertheless plays key roles in oogenesis and postimplantation development in mice (Molaro et al. 2020).

In contrast to spermatogenesis, oogenesis does not appear to involve dramatic chromatin changes analogous to the protamine transition. Yet maternal inheritance of certain histone variants is essential for embryonic viability and development (Martire and Banaszynski 2020). During oogenesis, chromatin undergoes chromosome condensation (Bogolyubova and Bogolyubov 2020), withstands double-stranded breaks during meiotic recombination, and survives a long meiotic arrest in mammals (Cheng et al. 2009; Lake and Hawley 2012; Carroll and Marangos 2013). In addition, histone variants may “mark” imprinted regions of the inherited maternal genome in embryos. Reprogramming of maternal genomes to match the epigenetic state of paternal genomes is also critical for fitness in many animals (Potok et al. 2013). Maternally deposited histone variants may also be crucial for the initial stages of embryogenesis, especially to mediate the protamine-to-histone transition of the paternal genome and posttranslational modifications of histones for zygotic genome activation. Despite these specialized chromatin requirements, very few chromatin innovations have been described for mammalian oogenesis, unlike for spermatogenesis. So far, only a few oocyte-specific variants, including some linker histone H1 variants, have been described (Martire and Banaszynski 2020; Talbert and Henikoff 2021). Although some H2A variants, including H2A.1, H2A.B, and macroH2A, are expressed during oogenesis, their functions, and their interactions with H2B variants remain uncharacterized. Our identification of two previously uncharacterized female germline-enriched histone variants—H2B.K and H2B.N—could thus reveal important insights into chromatin innovation and requirements during oogenesis. The oogenesis-expressed H2B variants (*H2B.1*, *H2B.K*, and *H2B.N*) are also detected in human embryos, suggesting the possibility of embryonic functions, which could be elucidated by future in vivo analyses.

The newly identified H2B variants also present some novel features that have not been previously observed in histones. Although H2B.K resembles RC H2B in its HFD, its highly diverged N-terminal tail includes a polyglutamine repeat and overall lower charge, suggesting it may confer different functionality and looser chromatin packing when incorporated into nucleosomes. Its near-ubiquitous presence across vertebrate genomes and strong sequence conservation motivates future functional studies. In contrast to H2B.K, H2B.N is dramatically different from RC H2B. For example, most H2B.N proteins have a significant C-terminal truncation that removes the αC domain, eliminating the important nucleosome acidic patch that mediates many other chromatin interactions (McGinty and Tan 2021). This feature is so unusual that that it raises the possibility of a nonnucleosomal function for H2B.N (like H2B.L), which could be revealed by biochemical and cytological analyses.

Our analyses not only reveal chromatin innovation in mammalian germlines but may also provide important clues for chromatin aberrations that can arise in cancer cells. For example, misexpression of other germline-specific histone variants and mutations in RC H2B can be detected in cancer cells (Bennett et al. 2019; Nacev et al. 2019; Bagert et al. 2021; Chew et al. 2021). Recent studies have identified H2B.W.2 as a potential driver gene in cervical cancer (Xu et al. 2021). Our phylogenomic analyses thus pave the way for future functional studies of H2B variants in gametogenesis and consequences of their misexpression in somatic cells, with implications for cancer and other diseases.

## Materials and Methods

### Identification of H2B Variants

To identify mammalian H2B variants we iteratively queried the assembled genomes of 18 mammals—human (*Homo sapiens*), mouse (*Mus musculus*), rat (*Rattus norvegicus*), guinea pig (*Cavia porcellus*), rabbit (*Oryctolagus cuniculus*), pig (*Sus scrofa*), sheep (*Ovis aries*), cow (*Bos taurus*), horse (*Equus caballus*), white rhinoceros (*Ceratotherium simum*), cat (*Felis catus*), dog (*Canis lupus familiaris*), panda (*Ailuropoda melanoleuca*), elephant (*Loxodonta africana*), armadillo (*Dasypus novemcinctus*), opossum (*Monodelphis domestica*), Tasmanian devil (*Sarcophilus harrisii*), and platypus (*Ornithorhynchus anatinus*), as well as a nonmammalian outgroup species, chicken (*Gallus gallus*) (supplementary table S1, Supplementary Material online). We used TBlastN (Altschul et al. 1990, 1997) on each species’ genome to perform a homology-based search starting with human H2B.W.1 (Q7Z2G1**)** (supplementary table S1, Supplementary Material online) as our query. We chose H2B.W.1 as a query sequence instead of an RC H2B to focus our search on more divergent H2B genes and because most mammalian genomes encode many near-identical RC H2B sequences. To ensure that we had not missed any divergent H2B homologs, we repeated our analyses using all H2B variants in this study as queries in TBlastN searches but did not retrieve additional hits (see supplementary file S1, Supplementary Material online).

To determine the age of *H2B.E*, we performed a TBlastN search for *H2B.E* in rodent and lagomorph genomes—western wild mouse (*Mus spretus*), ryukyu mouse (*Mus caroli*), shrew mouse (*Mus pahari*), wood mouse (*Apodemus sylvaticus*), deer mouse (*Peromyscus manuculatus*), short-tailed field vole (*Microtus agrestis*), prarie vole (*Microtus ochrogaster*), golden hamster (*Mesocricetus auratus*), chinese hamster (*Cricetulus griseus*), jerboa (*Jaculus jaculus*), kangaroo rat (*Dipodomys ordii*), brazilian guinea pig (*Cavia aperea*), squirrel (*Ictidomys tridecemlineatus*), alpine marmot (*Marmota marmota*), and pika (*Ochotona princeps*) (see supplementary file S2, Supplementary Material online).

To determine the age of *H2B.K*, we performed a TBlastN search for *H2B.K* in nonmammalian species—zebra finch (*Taeniopygia guttata*), western clawed frog (*Xenopus tropicalis*), coelacanth (*Latimeria chalumnae*), zebrafish (*Danio rerio*), elephant shark (*Callorhinchus milii*), lamprey (*Petromyzon marinus*), sea urchin (*Strongylocentrotus purpuratus*), and fruit fly (*Drosophila melanogaster*) (see supplementary file S3, Supplementary Material online).

H2B variant orthologs in 29 primates were identified using TBlastN analyses of NCBI’s nonredundant nucleotide collection (nr/nt) and whole-genome shotgun contigs (wgs) databases (see supplementary files S5 and S6, Supplementary Material online).

Once histone variants were identified, we used shared synteny (conserved genetic neighborhood) to identify putative orthologs in all representative mammalian genomes. We retrieved nucleotide sequences for all hits and their genomic neighborhoods, and recorded coordinates for syntenic analyses using the UCSC Genome Browser (Kent et al. 2002). For syntenic analyses (fig. 3*B* and supplementary figs. S3, S8, and S9, Supplementary Material online), two to three annotated genes on either side of each histone variant were identified (flanking genes) from mouse or human genomes. TBlastN searches using each flanking gene were performed to identify orthologs and therefore the syntenic regions in all selected mammalian genomes and chicken. In some genomes, the syntenic location was split between multiple scaffolds (double slashes in figures). *H2B.K* could not be identified in Tasmanian devil, likely because the syntenic region is split between two scaffolds: the intervening sequence may be missing from the assembly. Histones or flanking genes located on scaffolds labeled with Chr_UN were not included in our analyses.

Since RC H2Bs are present in numerous identical copies in mammalian genomes, we only used one copy of any identical RC H2Bs in our analyses. We used a copy of RC H2B that is present in six copies in the human genome (H2Bc4/H2Bc6/H2Bc7/H2Bc8/H2Bc10) and three copies in the mouse genome. Exons and introns in variants *H2B.W*, *H2B.K*, and *H2B.N* were annotated based either on protein alignments with closely related species or on Ensembl, RefSeq, or GenScan predictions. Since the N- and C-terminal residues of H2B.W orthologs show high divergence in mammals, our current annotations in nonprimate species may need to be revised with further experimental evidence. Pseudogenes were annotated based on disrupted ORFs or the presence of gene remnants as determined by a TBlastN search of the histone variant sequence against its syntenic regions (as in the case of *H2B.W* and *H2B.1*). In three cases, H2B variant copies were also found on the same chromosome immediately outside the syntenic location—one cow and sheep *H2B.W* variant and a horse *H2B.1* pseudogene. These are not annotated as other loci in figure 2*A* since they were found on the same chromosome as the ancestral gene near the syntenic region.

We used shared synteny, sequence similarity, and phylogenetic analyses (below) to classify ORFs and pseudogenes into H2B variant families (*H2B.E, H2B.L, H2B.1, H2B.W, H2B.K*, and *H2B.N*) (see supplementary file S8, Supplementary Material online).

### Phylogenetic Analyses

All protein and nucleotide alignments were performed using the MUSCLE algorithm (Edgar 2004) in Geneious Prime 2019.2.3 (https://www.geneious.com) and all phylogenies were generated using maximum-likelihood methods in PhyML (Guindon and Gascuel 2003; Guindon et al. 2010) with 100 bootstrap replicates. Since RC H2B are present in many near-identical copies in mammalian genomes, we used a random number generator to select two arbitrary copies of H2B from each species. Our protein phylogenies used alignments of either the HFD and αC domain, or the full-length sequences, with the Jones–Taylor–Thornton substitution model (Jones et al. 1992). Our nucleotide phylogenies used the HKY85 substitution model (supplementary figs. S11 and S12, Supplementary Material online). Pseudogenes were not included in any tree.

Sequences of *H2B.W* from mammals were analyzed for evidence of recombination using the GARD algorithm at datamonkey.org (Kosakovsky Pond et al. 2006).

### Calculating Rate of Protein Divergence for Histones

We used full-length protein sequences of all H2B variants to calculate pairwise identities between representative mammal orthologs (fig. 5*A* and supplementary table S2, Supplementary Material online). We used the human ortholog as a reference for all H2B variants, except H2B.L, which has been pseudogenized in humans; therefore, we used orangutan H2B.L as a reference sequence. Sequence divergence levels for H2A.P were obtained from a previous study (Molaro et al. 2018). We obtained median species divergence times from the TimeTree database (www.timetree.org) (Hedges et al. 2015).

### Analysis of Evolutionary Selective Pressures

We analyzed selective pressures on H2B variants in diverse mammals or in simian primates using the codeml algorithm from the PAML suite (Yang 1997) (supplementary tables S3 and S4, Supplementary Material online). For all tests, we generated codon alignments using MUSCLE (Edgar 2004), and manually adjusted them to improve alignments if needed. We also trimmed sequences to remove alignment gaps and segments of the sequence that were unique to only one species. We found no evidence of recombination for any of these alignments using the GARD algorithm at datamonkey.org (Kosakovsky Pond et al. 2006). We used the alignment to generate a tree using PhyML maximum-likelihood methods with the HKY85 substitution model (Guindon et al. 2010).

To test for gene-wide purifying selection (supplementary table S3, Supplementary Material online), we used codeml’s model 0, which assumes a single evolutionary rate for all lineages represented in the alignment. We compared likelihoods between model 0 with a fixed d*N*/d*S* value of 1 (neutral evolution) and model 0 with d*N*/d*S* estimated from the alignment. We determined statistical significance by comparing twice the difference in log-likelihoods between the two models with a χ^2^ distribution with 1 degree of freedom (Yang 1997).

To test whether a subset of residues evolves under positive selection (supplementary table S4, Supplementary Material online), we compared nested pairs of “NSsites” evolutionary models. We compared likelihoods between NSsites model 8 (where there are ten classes of codons with d*N*/d*S* between 0 and 1, and an eleventh class with d*N*/d*S* > 1) and either model 7 (which disallows d*N*/d*S* to be equal to or exceed 1) or model 8a (where the eleventh class has d*N*/d*S* fixed at 1). We determined statistically significance by comparing twice the difference in log-likelihoods between the models (M7 vs. M8 or M8 vs. M8a) to a χ^2^ distribution with the degrees of freedom reflecting the difference in number of parameters between the models being compared (Yang 1997). For alignments that showed statistically significant support for a subset of sites under positive selection, sites with a Bayes Empirical Bayes posterior probability >90% in M8 were classified as positively selected sites.

In addition to PAML, we used the FUBAR (Murrell et al. 2013) and BUSTED (Murrell et al. 2015) algorithms from datamonkey.org to estimate selection at each site or on the whole gene, respectively (supplementary table S4, Supplementary Material online).

### Logo Plots and Nucleosome Structure

Logo plots were generated using WebLogo (weblogo.berkeley.edu; Crooks et al. 2004) using one copy of each H2B variant or RC H2B protein sequences from each of the following species: sheep, dog, elephant, cow, bushbaby (*Otolemur garnettii*), mouse lemur (*Microcebus murinus*), and rhesus macaque (*Macaca mulatta*). These species were selected because they possess at least one intact copy of every H2B variant. We calculated a two-way JSD metric (Doud et al. 2015) at each amino acid position in the HFD and the αC domain as a quantitative estimate of conservation of each residue between each H2B variant and RC H2B. We also compared of all H2B variants together versus RC H2B as previously described (Molaro et al. 2018) to identify residues that differ between RC H2B and all H2B variants. This analysis did not reveal any residues common to all H2B variants and distinct from RC H2B.

We used Phyre2 (Kelley et al. 2015) to construct a homology model of the HFD of H2B variants. This software used existing H2B crystal structures to model the structure of human H2B variant protein sequences (or rhesus macaque for H2B.L). We used the Chimera software (Pettersen et al. 2004) to display the resultant predicted models with high confidence and highlighted residues of interest on a previously published human nucleosome structure (PDB:5y0c) (Arimura et al. 2018). The isoelectric point and charge for human H2B variants (supplementary fig. S7*C*, Supplementary Material online) were computed using Protpi (https://www.protpi.ch/).

### RNA-Seq Analysis

We analyzed publicly available transcriptome data from chicken, opossum, dog, pig, mouse, and human to approximately quantify expression of H2B variants in somatic and germline tissues (supplementary table S5, Supplementary Material online). We downloaded FASTQ files using NCBI’s SRA toolkit (https://www.ncbi.nlm.nih.gov/books/NBK158900), and mapped reads to same-species genome assemblies using the STAR mapper (Dobin et al. 2013). We used the “–outMultimapperOrder Random –outSAMmultNmax 1 –twopassMode Basic” options so that multiply mapping reads were assigned randomly to a single location. We then used genomic coordinates of each ORF and the BEDTools multicov tool (Quinlan and Hall 2010) to count reads overlapping each gene. We then used R (https://www.R-project.org/, last accessed January 28, 2022) to divide those counts by the total number of mapped reads in each sample in millions, followed by the size of each transcript in kb to obtain RPKM values. A previously published housekeeping gene, human C1orf43 (Eisenberg and Levanon 2013), was selected as a control (supplementary fig. S14, Supplementary Material online), and orthologs in other species were identified using Ensembl gene trees (Zerbino et al. 2018).

To search for stem loop sequences and poly(A) signals, we first extracted 600 bp of genomic sequence on each side of the stop codon of each H2B variant. We downloaded a model for the histone 3′-UTR stem loop (accession no. RF00032) from the RFAM database (Kalvari et al. 2021) searched for matches using the “cmsearch” algorithm (covariance model search) of the Infernal package (Nawrocki and Eddy 2013). For our analysis of poly(A) signals, we searched for exact matches to AATAAA or ATTAAA, the two most commonly found signal sequences in human transcripts (Beaudoing et al. 2000). This approach is somewhat limited, however. These short motifs will yield many false-positive matches, and previous analysis shows that many polyadenylated human transcripts have no recognizable signal sequence (Beaudoing et al. 2000). 

## Supplementary Material

Supplementary data are available at *Molecular Biology and Evolution* online.

## Supplementary Material

msac019_Supplementary_Data
