# Supplementary material for: Novel Classes and Evolutionary Turnover of Histone H2B Variants in the Mammalian Germline
Source: Mol Biol Evol. 2022 Jan 31;39(2):msac019. doi: 10.1093/molbev/msac019 (PMC8857922; doi:10.1093/molbev/msac019)
Supplement: msac019_Supplementary_Data [file msac019_supplementary_data.zip › Index of Supplementary materials.docx]

**Supplementary Figures**

Supplementary Figure S1. Phylogeny of mammalian H2B variants using the histone fold domain and αC domain.

Supplementary Figure S2. Mammalian H2B phylogeny using the histone fold domain.

Supplementary Figure S3. Synteny and sequence of H2B.E.

Supplementary Figure S4. Phylogeny of H2B.E in rodents.

Supplementary Figure S5. Alignment and expression of H2B.O in platypus.

Supplementary Figure S6. Phylogeny of full-length mammalian H2B.1 and RC H2B.

Supplementary Figure S7. Characteristics of H2B variants.

Supplementary Figure S8. Synteny of all germline-specific mammalian H2B variants.

Supplementary Figure S9. Phylogeny and synteny of H2B.K in vertebrates.

Supplementary Figure S10. Pseudogenization of H2B.L in some primates.

Supplementary Figure S11. Phylogeny of H2B.1 in primates.

Supplementary Figure S12. Phylogeny of H2B.W variants in primates.

Supplementary Figure S13. Positively selected residues of H2B.L and H2B.W.

Supplementary Figure S14. Expression of H2B variants in somatic and germline tissues of representative animals.

Supplementary Figure S15. Detecting presence of poly(A) and stem loop structures in H2B variants of representative animals.

**Supplementary Tables**

Supplementary Table S1. Genomic coordinates of all H2B variant genes in this study.

Supplementary Table S2. Divergence of H2Bs and H2A.P in mammals.

Supplementary Table S3. Results from purifying selection analyses (Mammals).

Supplementary Table S4. Detailed table of results from all analyses of positive selection.

Supplementary Table S5. List of SRA datasets used for RNA-seq analyses.

**Supplementary Datasets**

Supplementary Data S1. Alignments used to generate tree shown in Figure 1A, Supplementary Figure S1 and S2.

Supplementary Data S2. Alignments used to generate tree shown in Supplementary Figure S4.

Supplementary Data S3. Alignments used to generate tree shown in Supplementary Figure S9.

Supplementary Data S4. Alignments used to generate logos plots shown in Figure 2.

Supplementary Data S5. Alignments used to generate tree shown in Supplementary Figure S11.

Supplementary Data S6. Alignments used to generate tree shown in Supplementary Figure S12.

Supplementary Data S7. Alignments used for selection analyses in Figure 5B.

Supplementary Data S8. Sequence of all H2B variants in mammalian genomes used in this study.
